# Centralized Homologous Recombination Repair Testing in Metastatic Castration-Resistant Prostate Cancer: Real-World Data from a Multicenter Spanish Precision Oncology Program

**DOI:** 10.3390/cancers18101614

**Published:** 2026-05-16

**Authors:** Belén Caramelo, Pilar García-Berbel, Sofia del Carmen, Adriana Calapaqui, Luiz Corrêa, Lucia Martinez-Villaseñor, Marta Sotelo, Federico Rojo, Javier Gómez-Román, Ignacio Duran, Javier Freire

**Affiliations:** 1 Medical Oncology Department, Hospital Universitario Marqués de Valdecilla, 39008 Santander, Spain; belen.caramelo@scsalud.es (B.C.); marta.sotelo@scsalud.es (M.S.); 2 Instituto de Investigación Marqués de Valdecilla—IDIVAL, 39008 Santander, Spain; mpgarcia@idival.org (P.G.-B.); sofia.delcarmen@scsalud.es (S.d.C.); adrianakatherine.calapaqui@scsalud.es (A.C.); josejavier.gomez@scsalud.es (J.G.-R.); 3 Pathology Department, Hospital Universitario Marqués de Valdecilla, 39009 Santander, Spain; 4 Diagnostic Data Hub-DXD Hub, 08780 Barcelona, Spain; lggcorrea@dxd-hub.com; 5 Faculty of Medicine, Catholic University of Valencia San Vicente Mártir, 46001 Valencia, Spain; luciagberbel2@gmail.com; 6 Pathology Department, Fundación Jiménez Díaz, 28040 Madrid, Spain; frojo@fjd.es; 7 Department of Medical and Surgical Sciences, Faculty of Medicine, University of Cantabria, 39011 Santander, Spain

**Keywords:** *BRCA*, homologous recombination repair, PARP inhibitor, prostate cancer

## Abstract

**Background/Objectives:** Determining homologous recombination repair (HRR) status in metastatic castration-resistant prostate cancer (mCRPC) is essential to ensure access to targeted therapies, particularly PARP inhibitors. Yet, variability in testing access and analysis performance exists. This study evaluated feasibility and outcomes of a centralized HRR testing strategy in Spain for prostate cancer patients. **Methods:** A total of 1412 formalin-fixed paraffin-embedded (FFPE) tumor samples from mCRPC patients from 89 Spanish institutions within a centralized multicenter molecular testing program were analyzed using a standardized 38-gene-based next-generation sequencing (NGS) assay in a central laboratory (HRR OncoKit, Health in Code, Valencia, Spain), which included five clinically relevant HRR genes: *BRCA1*, *BRCA2*, *CHEK2*, *ATM*, and *CDK12*. **Results:** HRR gene pathogenic or likely pathogenic alterations were identified in 18% (CI 95% = 16–20) of the patients, with *BRCA2* being the most frequently altered gene (6%), followed by *ATM* (5%), *CDK12* (4%), *BRCA1* (2%), and *CHEK2* (1%). Eleven percent had variants of uncertain significance. Only 13% of the samples were rejected due to poor DNA quality, low tumor content or sample age exceeding 5 years, and 2% of the samples analyzed failed since the minimum library technical quality score was not achieved. The average turnaround time for results was 18 ± 3 days. **Conclusions:** Centralized HRR testing in mCRPC patients in Spain was feasible, efficient and reliable, identifying pathogenic alterations in 18% of the cases, similarly to the prevalence described in the literature. This testing approach facilitates precision medicine by improving the detection of actionable HRR alterations.

## Simple Summary

This study demonstrates that centralized genetic testing can effectively support precision medicine for patients with advanced prostate cancer across multiple hospitals. By analyzing tumor samples from a large national cohort using a standardized sequencing approach, we identified clinically relevant genetic alterations in 18% of patients. Our results show that this strategy provides reliable, high-quality results within a clinically useful timeframe, even when using routine diagnostic samples. Importantly, centralized testing ensures equal access to advanced molecular diagnostics and delivers consistent information to guide personalized treatment decisions. These findings highlight how large-scale, coordinated testing programs can be successfully integrated into everyday clinical practice, ultimately improving patient care and expanding access to targeted therapies.

## 1. Introduction

Homologous recombination repair (HRR) gene alterations are central to cancer development and progression. HRR preserves genomic stability by repairing DNA double-strand breaks. Mutations in HRR genes such as *BRCA1*, *BRCA2*, *ATM*, *CHEK2*, or *CDK12* impair repair capacity, driving genomic instability and cancer evolution [1]. Alterations in HRR-related genes have been identified across multiple tumor types, including ovarian, breast, pancreatic, and prostate cancers. In ovarian and breast cancer, *BRCA1/2* alterations may be present in up to 20–25% of cases, while HRR pathway alterations in pancreatic cancer have been reported in approximately 10–15% of patients, supporting the expanding role of DNA damage response-targeted therapies across solid tumors [2,3,4].

Exploiting this vulnerability, poly (ADP-ribose) polymerase inhibitors (PARPi) induce synthetic lethality in HRR-deficient cells. Normally, single-strand breaks are repaired via PARP-mediated base excision repair. When PARPi block PARP activity, these lesions convert into double-strand breaks. Healthy cells with intact HRR can repair them, but HRR-deficient tumor cells cannot, leading to lethal DNA damage. This selectivity allows PARPi to eliminate tumor cells while sparing normal tissue [3,5].

Prostate cancer is the second most frequently diagnosed cancer in men worldwide and the fifth leading cause of cancer-related death among men globally [6]. In prostate cancer, especially in advanced disease, HRR deficiencies are relatively frequent. Between 11–34% of metastatic castration-resistant prostate cancer (mCRPC) patients harbor such alterations, with *BRCA2* being the most prevalent, followed by *ATM*, *CHEK2*, and *BRCA1* [7,8,9,10]. These genes represent the most frequently altered and clinically actionable HRR biomarkers in mCRPC and account for the majority of alterations currently used to guide PARP inhibitor-based therapeutic strategies in routine clinical practice. Recent large genomic studies have shown that approximately 20–25% of advanced prostate cancers harbor alterations in DNA damage repair genes, with *BRCA2* alterations consistently associated with more aggressive clinical behavior, shorter survival, and increased sensitivity to PARP inhibition [11,12]. Reported prevalence varies across studies depending on whether testing is somatic or germline, the scope of genes analyzed, and population characteristics. HRR mutations are also prognostic and predictive biomarkers, influencing outcomes with PARPi [13,14,15,16,17].

Until recently, therapeutic options in mCRPC were limited. PARPi have expanded treatment strategies, with several agents approved. Olaparib is approved as monotherapy in HRR-mutated mCRPC and in combination with abiraterone as first-line therapy, irrespective of HRR status [18,19]. Rucaparib, approved by the FDA, is indicated for *BRCA1/2*-mutated mCRPC progressing after hormonal therapy and chemotherapy [20,21]. Talazoparib has been approved with enzalutamide for first-line mCRPC, and niraparib with abiraterone for *BRCA1/2*-mutated cases [22,23]. In Spain, however, current reimbursement and clinical implementation of PARPi are mainly restricted to patients harboring *BRCA1/2* alterations, reflecting the stronger and more consistent clinical benefit observed in this subgroup. These advances underscore the importance of identifying HRR alterations to select appropriate therapy.

Accordingly, guidelines recommend HRR testing in all patients with mCRPC [24]. However, implementation faces challenges: unequal access, lack of standardization, and variability in sample type (tumor vs. blood), mutation origin (somatic vs. germline), and panels employed [25]. Germline testing pathways, traditionally aimed at hereditary cancer risk assessment, may delay results and treatment. In contrast, therapy-oriented somatic testing of key actionable HRR genes provides faster results, better suited for real-time decision-making.

Despite its clinical value, HRR testing outcomes across studies remain inconsistent. Prevalence rates are highly variable, and most available evidence derives from clinical trials rather than real-world practice. Furthermore, trial reports often omit data on testing sensitivity, failure rates, and causes of unsuccessful analyses, limiting generalizability. A clearer understanding of HRR testing feasibility, performance, and mutation prevalence in unselected, real-world populations is therefore needed.

In this context, the present study aimed not only to estimate the prevalence of HRR alterations in a large multicenter Spanish cohort of mCRPC patients, but also to evaluate the feasibility, reliability, and real-world applicability of a centralized molecular testing strategy in routine clinical practice.

## 2. Materials and Methods

We conducted a national observational real-world study. From January-2022 to June-2024, a total of 1412 formalin-fixed paraffin-embedded (FFPE) tumor samples from mCRPC patients from 89 Spanish institutions were consecutively submitted to a centralized diagnostic platform (id.BRCA) created in collaboration with AstraZeneca. Inclusion criteria were patients with mCRPC pre-treated with ≥1 novel hormonal agent and who were eligible for a subsequent line of treatment (ECOG 0–1 and hemoglobin > 10 mg/dL). The minimum requirements for sample inclusion were a tumor cellularity of at least 30% and a sample age of less than 5 years [26]. Macrodissection was carried out to increase the tumor cellularity percentage in order to improve analytical sensitivity. Decalcified samples were not accepted. For library preparation following DNA extraction, a DNA Integrity Number greater than 3 and a concentration of at least 1.8 ng/µL (without concentration), yielding a total input of 25 ng, were required. DNA was extracted using the QIAamp DNA FFPE Advanced Kit FFPE DNA Isolation (Qiagen, Hilden, Germany), following the manufacturer’s protocol, which includes correction for formalin-induced deamination artifacts.

Samples were analysed using a standardized 38-gene based next-generation sequencing (NGS) assay in a central laboratory (HRR OncoKit, Health in Code, Valencia, Spain), which included five clinically relevant HRR genes: *BRCA1*, *BRCA2*, *CHEK2*, *ATM*, and *CDK12*. These genes were selected based on their prevalence in mCRPC and their clinical relevance, as *BRCA1/2* alterations currently constitute the main biomarkers associated with approved PARPi treatment in Spain. This panel was built under SureSelect XT HS (high-sensitivity) probe capture technology (Agilent, Santa Clara, CA, USA). The panel was automated using the Magnis Dx NGS Prep System (Agilent), with sequencing performed on the NextSeq 550 platform (Illumina, San Diego, CA, USA) at a minimum coverage of 200× unique molecular identifiers (UMIs) for precise molecular tagging. Bioinformatic analysis was conducted using the DataGenomics platform (Health in Code), enabling the detection of single nucleotide polymorphisms, small insertions and deletions, and copy number variations. To perform the clinical classification of genetic variants, the databases ClinVar, Varsome, BRCAShare, OncoKB, and BRCA Exchange were consulted during the week the report was issued [27,28,29,30,31,32]. The information was interpreted according to standardized criteria based on ACMG guidelines (Standards and Guidelines for the Interpretation of Sequence Variants) and the specific ENIGMA guidelines for *BRCA1/2* [33,34]. The following reference sequences from the NCBI Reference Sequence (RefSeq) database were used: *BRCA1* (NM_007294.4), *BRCA2* (NM_000059.4), *ATM* (NM_000051.4), *CHEK2* (NM_007194.4), and *CDK12* (NM_016507.4) [35].

This methodology may fail to identify exon-level deletions/insertions across entire genes when less than 50% tumor cellularity is present and when there are structural changes, mutations affecting splicing located in intronic regions not adjacent to exons, and mutations in regulatory sequences or epigenetic alterations. Although the assay allows for copy number analysis, its design limits interpretability by only detecting large gene deletions and not exon-level changes. While it can identify whole-gene deletions, particularly in *BRCA2*, it cannot detect focal or exon-level deletions that may be pathogenic in somatic contexts. Additionally, in tumors with homologous recombination deficiency, somatic whole-gene loss of heterozygosity may indicate genomic instability rather than biallelic inactivation, and without germline data, loss of heterozygosity events cannot be classified as pathogenic. Therefore, biallelic inactivation was not systematically evaluated due to the lack of exon-level resolution.

The data for result analysis were collected in collaboration with the DXDd hub (Diagnostic Data Hub, Pallejà, Spain). An artificial intelligence (AI) model was trained to automatically capture relevant information from the reports generated by the geneticists. This included data such as the hospital of origin, tumor cellularity, DNA extraction output, technical procedures, sample-related issues, and results from the final analysis of the genetic alterations.

The creation of relevant figures was carried out using Microsoft Excel, and the genetic lollipop mutation diagram plots were generated through the online program https://github.com/joiningdata/lollipops (accessed on 13 November 2025) [36].

### Statistical Analysis

MedCalc Statistical Software version 23.3.7 was used for the statistical analyses, which included the comparison of variant frequencies by genes and individuals, as well as the calculation of all general descriptive statistics associated with the technique. The prevalence of homologous recombination repair deficiency (HRRd) was calculated as the proportion of patients harboring pathogenic (PV) or likely pathogenic variants (LPV) among all patients with analyzable NGS results. VUS were analyzed separately and excluded from prevalence calculations, as only pathogenic and likely pathogenic variants were considered clinically actionable.

The results were stratified according to molecular findings and geographic distribution. Bonferroni correction was applied for multiple regional comparisons, considering statistical significance at *p* < 0.0033. Continuous variables were summarized using medians and interquartile ranges (IQRs), while categorical variables were expressed as frequencies and percentages. Only one sample per patient was included in the analysis.

## 3. Results

**Figure 1:**
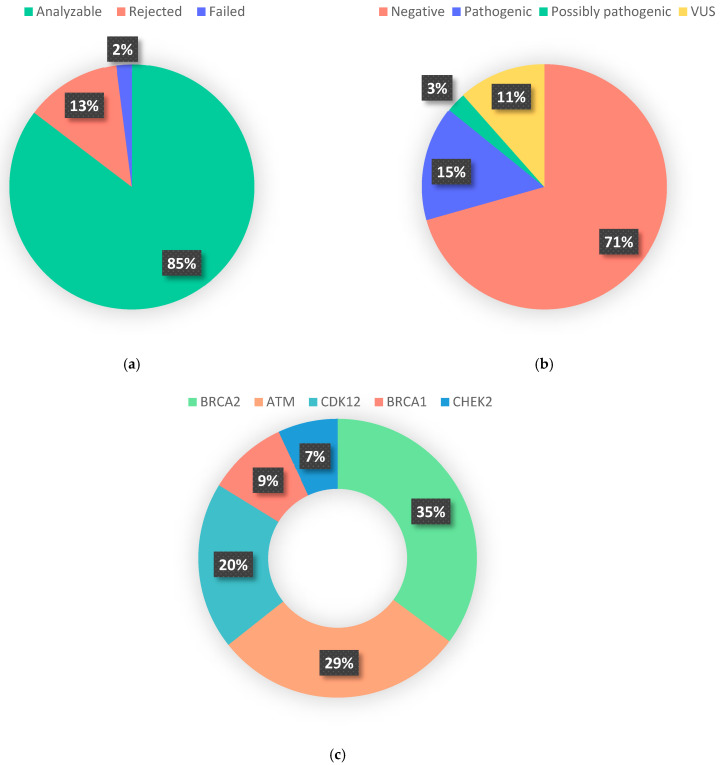
(**a**) Feasibility of HRR analysis. (**b**) HRR testing variants result. Variants of uncertain significance (VUS) (**c**) Proportion of pathogenic variants and likely pathogenic variants by gene.

Samples from 1412 patients with a median tumor purity of 60% (IQR 30–70) were received for analysis. Of these, 178 (13%) were rejected due to poor DNA quality (less than 1.8 ng/μL or DIN < 3), low tumor content (<30%), or sample age exceeding 5 years, in order to ensure the quality of the genetic material [26]. From the sequenced samples, 29 (2%) failed for not meeting the minimum library quality metrics (Figure 1a). The average turnaround time from the receipt of the sample at the hospital to the completion of the analysis was 18 ± 3 days. This period encompassed the morphological analysis, DNA extraction, library preparation, sequencing, and subsequent result analysis.

Twenty-nine percent (354) of the analyzed patients presented at least one alteration in the five selected genes of the HRR pathway, with 139 (11%) being VUS and 215 mutations classified as PV or LPV, resulting in an overall prevalence of HRRd of 18% (CI 95% = 16–20) (Figure 1b). Seventy-six *BRCA2* alterations were detected (6%), 63 (5%) in *ATM*, 43 (4%) in *CDK12*, 21 (2%) in *BRCA1*, and 15 (1%) in *CHEK2*. Proportion of the 5 analyzed genes is presented in Figure 1c. Forty-five patients (21% of the total mutated) presented alterations suggestive of “double-hit” events, defined as presence of two PVs or LPVs within the same HRR gene. These events were identified in 18 of 42 (43%) with *CDK12* alterations, 14 of 63 (22%) with *ATM* alterations, 11 of 76 (14%) with *BRCA2* alterations, and 2 of 21 (10%) with *BRCA1* alterations. Only three samples harbored concomitant pathogenic mutations in two different HRR genes.

**Figure 2:**
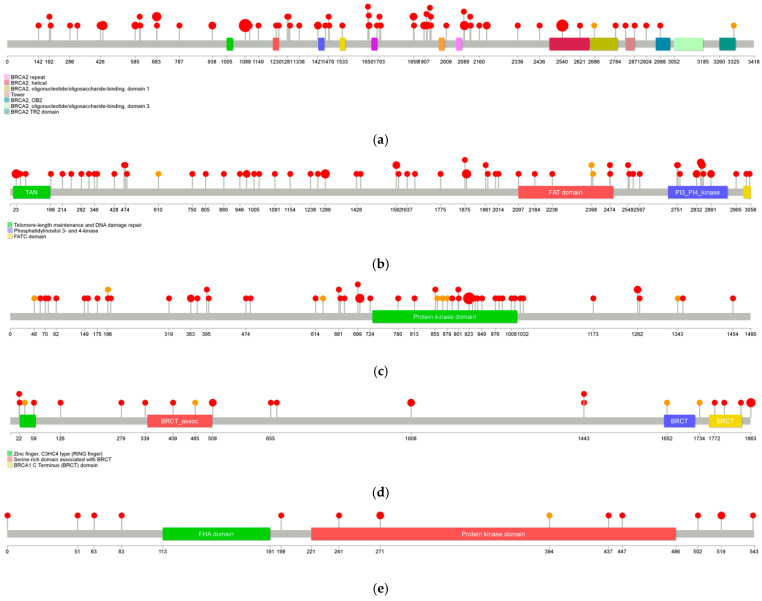
Pathogenic variants found per gene. Red: Pathogenic variants. Orange: Likely pathogenic variants. The size of each lollipop head reflects the frequency at which the mutation was detected. (**a**) *BRCA2*; (**b**) *ATM*; (**c**) *CDK12*; (**d**) *BRCA1*; (**e**) *CHEK2*.

The vast majority of the alterations found were point mutations, except in *BRCA2*, where deletions were more frequent (57%). The high frequency of mutations makes *BRCA2* the main molecular biomarker in this cohort. On the other hand, *ATM* showed notable mutational diversity, with a predominance of point mutations and a significant proportion of duplications and deletions. Although these alterations are associated with more variable clinical responses compared to *BRCA2*, they are still considered in the therapeutic selection of PARPi. *CDK12*, in turn, demonstrated a high variability and diversity of mutation types detected across HRR genes, with numerous deletions and point mutations identified. Cases suggestive of “double-hit” were found in 43% of patients with *CDK12* HRR mutations, although true biallelic loss or complete functional inactivation could not be confirmed with this assay. Within *BRCA1*, several variants were identified across all categories: point mutations, deletions, duplications, and insertions. Finally, mainly point mutations were observed in *CHEK2*, with the T519M, A502P, and E437K variants being the most common (Figure 2). All the alterations identified during the analysis are thoroughly documented and can be reviewed in detail in the Supplementary Material (Table S1).

**Figure 3:**
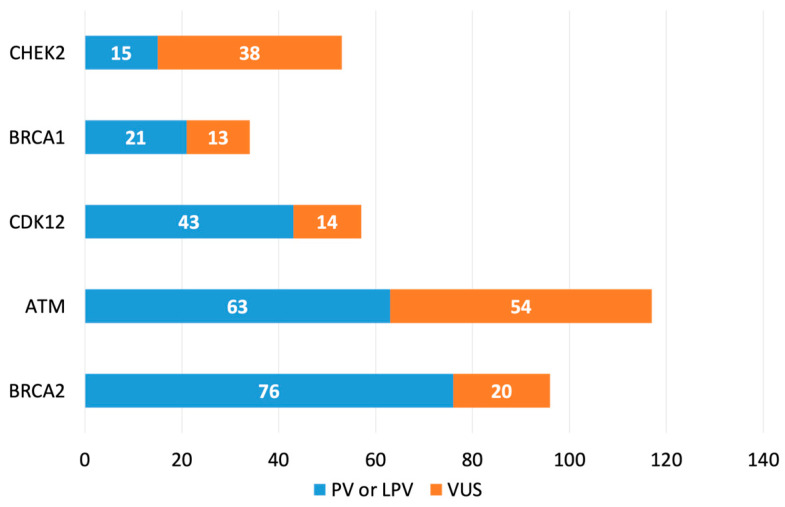
Variants of uncertain significance (VUS) per gene compared with pathogenic variants (PV) and likely pathogenic variants (LPV).

Regarding VUS, it is noteworthy that although nearly half of the PV or LPV detected alterations were located in *BRCA1* and *BRCA2*, approximately 80% of the VUS corresponded to genes other than these two. This finding highlights the current limited understanding of HRR mechanisms beyond *BRCA*. *ATM* (39%) and *CHEK2* (27%) were the most frequently implicated, followed by *BRCA2* (15%), *CDK12* (10%), and *BRCA1* (9%) (Figure 3).

In addition, analysis of the allelic frequencies of the detected alterations showed that 14% of cases exhibited a variant allele frequency (VAF) of 40% or higher, which may be suggestive of a germline origin and could therefore warrant genetic counseling and dedicated germline testing for confirmation.

**Figure 4:**
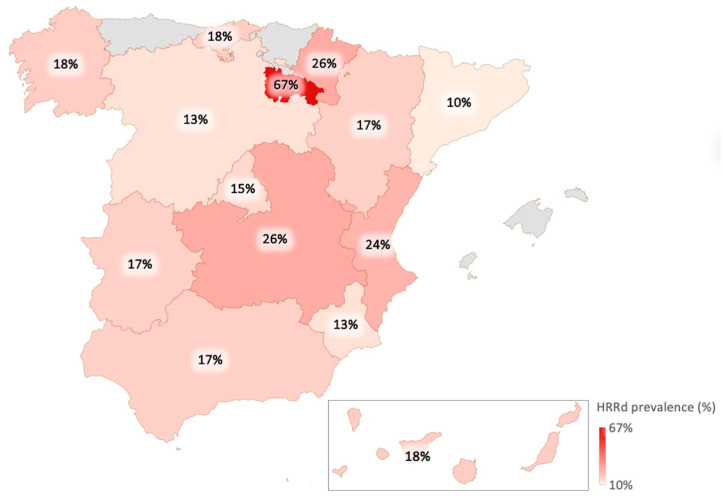
Map of HRRd prevalence by geographical distribution in Spain.

Finally, the analysis of alterations by region revealed variations in geographic distribution, with mutational prevalence rates ranging from 13% to 67% across the different regions of Spain (Figure 4).

Regions with the highest HRRd rates were La Rioja, 67% (95% CI = 22–96%); Castile-La Mancha, 26% (95% CI = 19–35%); and Navarre, 26% (95% CI = 9–51%) (Table 1).

Although the associations observed in La Rioja and Castile-La Mancha initially reached statistical significance (*p* = 0.0018 and *p* = 0.0111, respectively), only the association observed in La Rioja remained statistically significant after Bonferroni correction for 14 regional comparisons (adjusted significance threshold *p* < 0.0036). However, this finding should be interpreted with caution given the low sample size from this region. Therefore, these analyses should be considered exploratory, and no causal inference can be drawn, as establishing causality would require more extensive analyses in a larger cohort.

## 4. Discussion

Determining HRR status in mCRPC is essential to ensure access to targeted therapies, particularly PARP inhibitors. While most published studies focus on prevalence and therapeutic implications, less is known about the feasibility of routine testing, failure rates, and real-world challenges. Much of the available evidence derives from clinical trials, where reporting on test failures is inconsistent. In PROfound, for instance, HRR testing showed a 31% failure rate, largely due to DNA extraction problems and archived material quality, with success rates higher in newly collected than archival samples (64% vs. 57%), with a significantly lower success rates in samples older than five years (50% vs. 68% for samples collected within the past year) [26]. Bone biopsies also performed poorly because decalcification procedures degrade nucleic acids (43% vs. 75% for other tissues) [13]. Similar failure rates were observed in GALAHAD, PROpel, and TALAPRO-2 (26–32%) [22,37,38]. In contrast, TOPARP-B, which used a more focused NGS panel optimized for FFPE tissue, reported only 15% failure [39]. Observational studies such as PROREPAIR-B and CAPTURE did not systematically report failures, limiting understanding of pre-analytical and analytical barriers to HRR testing [1,40].

Our study showed a 13% rejection rate, primarily related to sample quality, and only 2% analytical failure. These results emphasize the importance of rigorous pre-analytical selection and specialized panels. The Agilent SureSelect XT HS platform, specifically tailored to FFPE samples, demonstrated excellent reliability and efficiency, consistent with TOPARP-B findings [39]. The hybrid capture method further enhanced coverage, particularly for structural variants and loss of heterozygosity, both critical for HRR deficiency assessment. Therapy-driven targeted panels, by focusing on clinically relevant HRR genes, not only reduced turnaround time (18 days vs. ~6 weeks for hereditary reports) but also increased the proportion of patients identified as eligible for PARP inhibitors [41].

The prevalence of PV or LPV (18%) aligns with prior reports but highlights variability across studies. Clinical trial data show a wide range prevalence across different studies (between 11–68%), which may be explained by biases arising from the varying selection criteria and whether somatic testing, in addition to germline testing, is included [19,20,21,22,23,26,39]. Real-world databases further illustrate this variability, with prevalence of 25% (*n* = 487, somatic + germline) in Clinico-Genomic Database and 11% (*n* = 3270, somatic only) in the American Association for Cancer Research Project Genomics Evidence Neoplasia Information Exchange [7]. Additionally, some studies include VUS within HRR alteration estimates, which may further contribute to discrepancies in reported prevalence rates. In the present study, only pathogenic and likely pathogenic variants were considered in prevalence calculations to provide a more clinically meaningful estimate of actionable alterations, particularly given that most VUS are eventually reclassified as benign or likely benign [42,43]. Differences in methodology, gene panels, and population characteristics make cross-study comparisons difficult, underscoring the need for standardized approaches.

An interesting finding was the presence of alterations suggestive of “double-hit” alterations in 21% of mutated cases, most frequently in *CDK12* and *ATM*. Although their clinical implications are still under investigation, these events may indicate biallelic inactivation and a more aggressive tumor phenotype [44,45]. Conversely, concomitant mutations in different HRR genes were rare (*n* = 3), suggesting independent rather than syndromic events.

Variant interpretation remains a challenge. In our study, 80% of VUS occurred outside *BRCA1/2*, most frequently in *ATM* and *CHEK2*. This highlights the limited understanding of non-*BRCA* HRR biology and the risk of under-recognizing patients who could benefit from PARP inhibitors. Homologous recombination deficiency (HRD) genomic signatures have been proposed as surrogates for pathogenicity, as in ovarian or triple-negative breast cancer, where HRD scores predict PARPi benefit beyond *BRCA* mutations [46,47]. However, in prostate cancer, HRD scoring has not demonstrated incremental predictive value over direct mutation profiling. Evidence from PROfound, TRITON-2, and TOPARP-B consistently shows that PARPi efficacy is mainly driven by deleterious *BRCA2* alterations, while outcomes for other HRR genes are less consistent [20,26,39]. Accordingly, current guidelines recommend targeted sequencing of *BRCA1*, *BRCA2*, *ATM*, *CHEK2*, and *CDK12* rather than broader HRD assays. Our results support this focused, therapy-driven approach.

Geographic heterogeneity in prevalence was also observed across Spanish regions. Although statistically significant differences were initially identified in La Rioja and Castile-La Mancha, only the association observed in La Rioja remained significant after correction for multiple comparisons. Nevertheless, regional analyses should be considered exploratory, particularly given the limited sample size in some regions. In addition, causal interpretation cannot be made due to possible biases such as population differences, founder effects, sample variability, or unequal access to testing.

Despite guideline recommendations, uptake of HRR testing in routine practice is still suboptimal. Data from 2013–2018 show that only 13% of mCRPC patients underwent testing, rising to 38% by 2020, but still far from universal [48,49]. Barriers include reliance on archived tissue, which may be degraded; difficulties in obtaining high-quality DNA from bone biopsies; and logistical challenges in molecular testing pathways. Our centralized model, with strict pre-analytical controls and optimized technology, demonstrates that these barriers can be overcome, delivering reliable results within clinically relevant timeframes.

This study has some limitations. Only five genes, although they account for 85% of the total HRR mutation, were analyzed, so conclusions cannot be drawn regarding other genes reported in other trials. As this study was conducted within a centralized and fully anonymized platform, we did not have access to specific pre-analytical details such as sample type (biopsy vs. resection) or tumor location (primary vs. metastatic). Additionally, due to the centralized nature of the platform, germline validation was not feasible, so only FFPE tumor samples were analyzed, limiting distinction between somatic and germline mutations and preventing copy number variations confirmation. The use of archival FFPE samples may have introduced variability in DNA quality, potentially affecting the success of the analysis. These factors highlight the need for further research using comprehensive genomic approaches, germline testing, and balanced regional representation to validate and expand these findings.

## 5. Conclusions

In summary, our real-world experience highlights the feasibility and efficiency of centralized HRR testing using therapy-driven targeted panels is feasible and compatible with routine clinical decision-making, with low analytical failure rates and clinically acceptable turnaround times. Prevalence of HRR alterations was consistent with prior reports, with *BRCA2* remaining the predominant biomarker. In addition, alterations suggestive of “double-hit” events and the distribution of VUS were characterized within this large multicenter cohort.

Future efforts should focus on expanding equitable access to molecular testing, integrating somatic and germline analyses, refining HRR panel composition, and improving the interpretation of non-*BRCA* alterations and VUS. Correlation of molecular findings with treatment outcomes and prospective real-world clinical data will be essential to optimize patient selection and further consolidate precision oncology strategies in mCRPC.

## Figures and Tables

**Table 1:** Table of HRRd prevalence by geographical distribution in Spain.

Region	Analyzed Samples	LPV or PV	HRRd Prevalence (%)	CI 95%	*p*
Andalusia	6	1	16.66	0.42–64.12	0.9418
Aragon	47	8	17.02	7.65–30.81	0.8888
Canary Islands	90	16	17.77	10.52–27.25	0.9941
Cantabria	74	13	17.56	9.70–28.16	0.9570
Castile-Leon	229	30	13.1	9.02–18.17	0.0630
Castile-La Mancha	129	34	26.35	18.99–34.83	0.0111
Catalonia	39	4	10.25	2.86–24.21	0.2177
Extremadura	53	9	16.98	8.07–29.80	0.8760
Galicia	130	23	17.69	11.56–25.35	0.9738
La Rioja	6	4	66.66	22.27–95.67	0.0018
Madrid	165	24	14.54	9.54–20.86	0.2736
Murcia	72	9	12.5	5.88–22.41	0.2397
Navarre	19	5	26.31	9.14–51.197	0.3322
Valencian Community	146	35	23.97	17.30–31.73	0.0513

LPV: Likely pathogenic variants; PV: Pathogenic variants; HRRd: Homologous recombination repair deficiency; CI: Confidence interval.

## Data Availability

The original contributions presented in this study are included in the article and Supplementary Materials. Further inquiries can be directed to the corresponding authors.

## Supplementary Materials

The following supporting information can be downloaded at https://www.mdpi.com/article/10.3390/cancers18101614/s1, Table S1: Comprehensive list of identified alterations.

## Abbreviations

The following abbreviations are used in this manuscript:

AI	Artificial intelligence
FFPE	Formalin-fixed paraffin-embedded
IQR	Interquartile range
HRD	Homologous recombination deficiency
HRR	Homologous recombination repair
HRRd	Homologous recombination repair deficiency
LPV	Likely pathogenic variant
mCRPC	Metastatic castration-resistant prostate cancer
PARPi	Polymerase inhibitors
PV	Pathogenic variant
NGS	Next-generation sequencing
VAF	Variant allele frequency
VUS	Variants of uncertain significance
