# Supplementary material for: Centralized Homologous Recombination Repair Testing in Metastatic Castration-Resistant Prostate Cancer: Real-World Data from a Multicenter Spanish Precision Oncology Program
Source: Cancers (Basel). 2026 May 16;18(10):1614. doi: 10.3390/cancers18101614 (PMC13204402; doi:10.3390/cancers18101614)
Supplement: Supplementary file 1 [file cancers-18-01614-s001.zip › cancers-4287395-supplementary.pdf]

Table S1: Comprehensive list of identified alterations.

GENE	ACMG Variant Classification	Coding DNA position	Protein Position	No. of cases observed
ATM	Pathogenic	1038del	I346MfsX3	1
ATM	Pathogenic	1071del	F357LfsX33	1
ATM	Pathogenic	118_125del	I40SfsX20	1
ATM	Pathogenic	1282_1285del	P428TfsX8	1
ATM	Pathogenic	1402_1403del	K468EfsX18	1
ATM	Pathogenic	1420del	S474AfsX8	1
ATM	Pathogenic	1439_1442del	L480X	1
ATM	Pathogenic	185+1G>T	?	1
ATM	Pathogenic	2250G>A	K750K	1
ATM	Pathogenic	2413C>T	R805X	1
ATM	Pathogenic	2639-2A>T	?	1
ATM	Pathogenic	2838+1G>T	?	1
ATM	Pathogenic	2921+1G>A	?	2
ATM	Pathogenic	3014dup	N1005KfsX5	1
ATM	Pathogenic	3077+2T>G	?	1
ATM	Pathogenic	3273_3277del	E1091DfsX3	1
ATM	Pathogenic	3461T>A	L1154X	1
ATM	Pathogenic	3713dup	L1238FfsX8	1
ATM	Pathogenic	3802del	V1268X	1
ATM	Pathogenic	3894dup	A1299CfsX3	3
ATM	Pathogenic	4282_4295del	E1428LfsX2	1
ATM	Pathogenic	4335_4338del	F1445LfsX5	1
ATM	Pathogenic	4777-5_4795del	?	1
ATM	Pathogenic	4800_4803del	S1601FfsX7	1
ATM	Pathogenic	4910-22_5005+8del	?	1
ATM	Pathogenic	496+1del	?	1
ATM	Pathogenic	5005G>T	E1669X	1
ATM	Pathogenic	5324del	L1775X	1
ATM	Pathogenic	5623C>T	R1875X	1
ATM	Pathogenic	5644C>T	R1882X	3
ATM	Pathogenic	5883T>G	Y1961X	1
ATM	Pathogenic	5908C>T	Q1970X	1
ATM	Pathogenic	6004C>T	Q2002X	1
ATM	Pathogenic	6040G>T	E2014X	1
ATM	Pathogenic	6289G>T	E2097X	1
ATM	Pathogenic	640dup	S214FfsX40	1
ATM	Pathogenic	6490G>T	E2164X	1

ATM	Pathogenic	6706G>T	E2236X	1
ATM	Pathogenic	67C>T	R23X	3
ATM	Pathogenic	7421T>G	L2474X	1
ATM	Pathogenic	7424T>A	L2475X	1
ATM	Pathogenic	748C>T	R250X	1
ATM	Pathogenic	7646C>A	S2549X	1
ATM	Pathogenic	7660del	H2554IfsX10	1
ATM	Pathogenic	7708G>T	E2570X	1
ATM	Pathogenic	7789-1G>A	?	1
ATM	Pathogenic	8251_8254del	T2751SfsX54	1
ATM	Pathogenic	8264_8268del	Y2755CfsX1	1
ATM	Pathogenic	8287C>T	R2763X	1
ATM	Pathogenic	8494C>T	R2832C	2
ATM	Pathogenic	8504_8577dup	S2860AfsX22	1
ATM	Pathogenic	8545C>T	R2849X	2
ATM	Pathogenic	8560C>T	R2854C	2
ATM	Pathogenic	8671+1G>A	?	1
ATM	Pathogenic	8671+5G>C	?	1
ATM	Pathogenic	875C>T	P292L	1
ATM	Pathogenic	8988-1G>C	?	1
ATM	Pathogenic	9109C>T	Q3037X	1
ATM	Pathogenic	9169_*10del	?	1
ATM	Pathogenic	967A>G	I323V	1
ATM	VUS	5934A>C	E1978D	1
ATM	VUS	1516G>T	G506C	1
ATM	VUS	1595G>A	C532Y	2
ATM	VUS	1607+4del	?	1
ATM	VUS	2051A>G	Q684R	1
ATM	VUS	2056C>T	L686F	1
ATM	VUS	2353C>T	R785C	1
ATM	VUS	2414G>A	R805Q	1
ATM	VUS	2466+43_2466+44insT	?	1
ATM	VUS	2698A>G	M900V	1
ATM	VUS	2770C>T	R924W	1
ATM	VUS	295A>G	S99G	1
ATM	VUS	3402+7T>G	?	1
ATM	VUS	3469_3471del	L1157Del	1
ATM	VUS	3505G>A	E1169K	1
ATM	VUS	3586A>G	K1196E	1
ATM	VUS	3834C>A	D1278E	1
ATM	VUS	4148C>T	S1383L	3
ATM	VUS	4151A>G	H1384R	1

ATM	VUS	4424A>G	Y1475C	1
ATM	VUS	4853G>A	R1618Q	1
ATM	VUS	4944_4946del	V1649Del	1
ATM	VUS	5185G>C	V1729L	2
ATM	VUS	5189G>A	R1730Q	1
ATM	VUS	5497-4C>G	?	1
ATM	VUS	5639C>T	T1880M	1
ATM	VUS	5812T>G	Y1938D	1
ATM	VUS	5840C>G	S1947C	1
ATM	VUS	5876A>G	E1959G	1
ATM	VUS	5880C>G	I1960M	1
ATM	VUS	6016T>A	L2006I	1
ATM	VUS	6024C>G	I2008M	1
ATM	VUS	610G>A	G204R	2
ATM	VUS	6213G>T	L2071F	1
ATM	VUS	6239A>G	Y2080C	1
ATM	VUS	6386A>G	Y2129C	1
ATM	VUS	6604T>G	Y2202D	1
ATM	VUS	6848C>T	S2283L	2
ATM	VUS	712A>G	I238V	1
ATM	VUS	7187C>G	T2396S	1
ATM	VUS	7284G>C	R2428S	1
ATM	VUS	7375C>G	R2459G	2
ATM	VUS	7793G>A	R2598Q	1
ATM	VUS	7836_7838de	R2613Del	1
ATM	VUS	7871G>C	C2624S	1
ATM	VUS	8143C>A	L2715I	1
ATM	VUS	8362C>T	H2788Y	1
ATM	VUS	8560C>T	R2854C	1
ATM	VUS	8560C>T	R2854C	3
ATM	VUS	8600G>T	G2867V	1
ATM	VUS	8666A>G	D2889G	1
ATM	VUS	8810T>C	V2937A	1
ATM	VUS	8921C>T	P2974L	1
ATM	VUS	956T>C	L319P	1
ATM	VUS	971G>A	S324N	1
ATM	VUS	984_986del	S328Del	1
ATM	Likely pathogenic	1828A>T	K610X	1
ATM	Likely pathogenic	4777-5_4795del	?	1

ATM	Likely pathogenic	7191dup	Y2398IfsX5	1
ATM	Likely pathogenic	7212del	Y2404X	1
ATM	VUS	6491A>T	E2164V	1
BRCA1	Pathogenic	1016del	K339Rfs*2	1
BRCA1	Pathogenic	107C>A	E2164V	1
BRCA1	Pathogenic	1525_1535del	T509SfsX3	2
BRCA1	Pathogenic	175del	S59HfsX10	1
BRCA1	Pathogenic	1961dup	Y655VfsX18	1
BRCA1	Pathogenic	2008G>T	E670X	1
BRCA1	Pathogenic	3023_3024insTTTGGCCCTCTG TTTCTACCTAGTTCAAT	M1008IfsX26	2
BRCA1	Pathogenic	376C>T	Q126X	1
BRCA1	Pathogenic	4327C>T	R1443X	1
BRCA1	Pathogenic	4328_4683del	F1443SfsX13	1
BRCA1	Pathogenic	5215+1_5215+20del	?	1
BRCA1	Pathogenic	5314C>T	R1772X	1
BRCA1	Pathogenic	5387C>A	S1796X	1
BRCA1	Pathogenic	5592_*104del	?	3
BRCA1	Pathogenic	65T>C	L22S	1
BRCA1	Pathogenic	68_69delAG	E23VfsX17	1
BRCA1	Pathogenic	835del	H279MfsX19	1
BRCA1	VUS	1205A>T	E402V	1
BRCA1	VUS	1243G>T	V415F	1
BRCA1	VUS	1877T>C	V626A	1
BRCA1	VUS	2692A>G	K898E	1
BRCA1	VUS	301+6T>C	?	1
BRCA1	VUS	3047A>G	N1016S	1
BRCA1	VUS	3259G>A	G1087R	1
BRCA1	VUS	3276G>C	E1092D	1
BRCA1	VUS	3442G>C	E1148Q	1
BRCA1	VUS	4046C>G	T1349R	1
BRCA1	VUS	4168G>T	D1390Y	1
BRCA1	VUS	4972A>C	T1658P	1
BRCA1	VUS	6521T>G	V2174G	1
BRCA1	VUS	793T>A	S265T	1
BRCA1	Likely pathogenic	107C>A	S36Y	1
BRCA1	Likely pathogenic	1394A>G	Y465C	1
BRCA1	Likely pathogenic	4956G>A	M1652I	1

BRCA1	Likely pathogenic	5202T>G	F1734L	1
BRCA1	Pathogenic	1225_1615del	V409RfsX7	1
BRCA2	Pathogenic	1276_1522del	K426X	2
BRCA2	Pathogenic	1310_1313del	K437IfsX22	3
BRCA2	Pathogenic	1754del	K585RfsX29	2
BRCA2	Pathogenic	1813del	I605YfsX9	1
BRCA2	Pathogenic	1821_1822insA	D608RfsX8	1
BRCA2	Pathogenic	2043_2098del	S683YfsX6	3
BRCA2	Pathogenic	2050C>T	Q684X	1
BRCA2	Pathogenic	2359A>T	R787X	1
BRCA2	Pathogenic	2808_2811del	A938PfsX21	2
BRCA2	Pathogenic	2957_3323dup	A1109X	1
BRCA2	Pathogenic	3264dup	Q1089SfsX10	6
BRCA2	Pathogenic	3446_3458del	M1149RfsX15	1
BRCA2	Pathogenic	3446_4364dup	E1455DfsX9	1
BRCA2	Pathogenic	3689del	S1230LfsX9	1
BRCA2	Pathogenic	3725del	S1242MfsX17	1
BRCA2	Pathogenic	3841A>T	K1281X	1
BRCA2	Pathogenic	3860dup	N1287KfsX2	1
BRCA2	Pathogenic	3878_3932del	I1293KfsX24	1
BRCA2	Pathogenic	4005dup	F1336IfsX2	1
BRCA2	Pathogenic	425G>A	S142N	1
BRCA2	Pathogenic	4262_4263del	F1421X	2
BRCA2	Pathogenic	4407_5261del	I1470_D1754Del	1
BRCA2	Pathogenic	4417_4481del	N1473CfsX19	1
BRCA2	Pathogenic	4483_4595dup	K1533SfsX9	1
BRCA2	Pathogenic	4948dup	S1650KfsX16	1
BRCA2	Pathogenic	4960_4963del	C1654TfsX15	1
BRCA2	Pathogenic	4964dup	Y1655X	1
BRCA2	Pathogenic	4965C>A	Y1655X	1
BRCA2	Pathogenic	5073dup	W1692MfsX3	1
BRCA2	Pathogenic	5107G>T	E1703X	1
BRCA2	Pathogenic	5126_5674del	D1709_A1891Del	1
BRCA2	Pathogenic	5576_5579del	I1859KfsX3	2
BRCA2	Pathogenic	5580_5583del	K1861X	1
BRCA2	Pathogenic	5678_5760dup	F1921VfsX16	2
BRCA2	Pathogenic	5720_5723del	S1907X	2
BRCA2	Pathogenic	574_575del	M192VfsX13	1
BRCA2	Pathogenic	5751_5770del	H1918SfsX3	1
BRCA2	Pathogenic	5800C>T	Q1934X	1
BRCA2	Pathogenic	5812_6514del	G1938HfsX19	1

BRCA2	Pathogenic	5817_6173del	L1939_G2057Del	1
BRCA2	Pathogenic	593T>G	L198X	1
BRCA2	Pathogenic	6024dup	Q2009AfsX9	1
BRCA2	Pathogenic	6265G>T	E2089X	1
BRCA2	Pathogenic	6275_6276del	L2092PfsX7	3
BRCA2	Pathogenic	6353_6615del	V2118EfsX19	1
BRCA2	Pathogenic	6361G>T	E2121X	1
BRCA2	Pathogenic	6478_6802del	Q2160EfsX12	1
BRCA2	Pathogenic	6582_6785del	I2194_E2261Del	2
BRCA2	Pathogenic	7007G>A	R2336H	1
BRCA2	Pathogenic	7307del	N2436TfsX33	1
BRCA2	Pathogenic	7618-5_7705del	?	5
BRCA2	Pathogenic	7861del	Y2621IfsX27	1
BRCA2	Pathogenic	8351G>A	R2784Q	1
BRCA2	Pathogenic	857C>A	S286X	1
BRCA2	Pathogenic	8611G>T	E2871X	1
BRCA2	Pathogenic	8771_8850del	E2924GfsX6	1
BRCA2	Pathogenic	8953+1_9102del	?	1
BRCA2	Pathogenic	958del	L320YfsX4	1
BRCA2	VUS	1813A>T	I605L	1
BRCA2	VUS	2159_2296del	D720_N765Del	1
BRCA2	VUS	2981C>T	A994V	1
BRCA2	VUS	3055C>G	L1019V	1
BRCA2	VUS	3503T>C	M1168T	1
BRCA2	VUS	4008_4103del	F1336_K1367Del	1
BRCA2	VUS	4045A>G	I1349V	1
BRCA2	VUS	520C>T	R174C	1
BRCA2	VUS	5660C>T	T1887M	1
BRCA2	VUS	5729A>T	N1910I	2
BRCA2	VUS	681+5_681+8del	?	1
BRCA2	VUS	7007+3_7007+11del	?	2
BRCA2	VUS	8482A>G	I2828V	2
BRCA2	VUS	8518A>G	I2840V	2
BRCA2	VUS	8633-4T>A	?	2
BRCA2	VUS	8754+3G>A	?	1
BRCA2	VUS	9053G>A	S3018N	1
BRCA2	VUS	9086C>T	A3029V	1
BRCA2	VUS	9333_9335del	E3111Del	1
BRCA2	Likely pathogenic	8055_8056dup	L2686HfsX9	1
BRCA2	Likely pathogenic	9973_10105del	F3325LfsX14	1

BRCA2	Pathogenic	8485C>T	Q2829X	1
CDK12	Pathogenic	1088C>G	S363X	2
CDK12	Pathogenic	1125del	S376VfsX60	1
CDK12	Pathogenic	1184_1185del	L395RfsX3	1
CDK12	Pathogenic	1195_1204del	L399KfsX34	1
CDK12	Pathogenic	1421dup	N474KfsX12	1
CDK12	Pathogenic	1445dup	L483VfsX3	1
CDK12	Pathogenic	177del	A60QfsX32	1
CDK12	Pathogenic	1839_1840del	S614CfsX26	1
CDK12	Pathogenic	1981_1982del	R661DfsX38	1
CDK12	Pathogenic	1991dup	H664QfsX36	1
CDK12	Pathogenic	2015del	P672LfsX81	1
CDK12	Pathogenic	2095A>T	K699X	1
CDK12	Pathogenic	209del	L70WfsX22	1
CDK12	Pathogenic	2108+1G>T	?	2
CDK12	Pathogenic	2109-1G>T	?	1
CDK12	Pathogenic	2170_2174del	V724QfsX3	1
CDK12	Pathogenic	227del	I76TfsX16	1
CDK12	Pathogenic	2339_2355del	Q780RfsX3	1
CDK12	Pathogenic	2437_2460dup	F813_L820Dup	1
CDK12	Pathogenic	2564del	F855SfsX13	1
CDK12	Pathogenic	2667-6T>G	?	1
CDK12	Pathogenic	2703C>G	Y901X	1
CDK12	Pathogenic	2704C>T	R902X	1
CDK12	Pathogenic	273_309del	D92GfsX20	1
CDK12	Pathogenic	2768+1G>A	?	2
CDK12	Pathogenic	2769-1G>C	?	2
CDK12	Pathogenic	2769-3T>G	?	1
CDK12	Pathogenic	2789del	F930SfsX16	1
CDK12	Pathogenic	2803_2804del	I935FfsX19	1
CDK12	Pathogenic	2819del	L940RfsX6	1
CDK12	Pathogenic	2847-2A>C	?	1
CDK12	Pathogenic	2926_2939del	K976AfsX15	1
CDK12	Pathogenic	2969del	P990LfsX12	1
CDK12	Pathogenic	3022C>T	R1008W	1
CDK12	Pathogenic	3044_3080del	L1015QfsX30	1
CDK12	Pathogenic	3075del	S1026AfsX31	1
CDK12	Pathogenic	3096-2A>T	?	1
CDK12	Pathogenic	3784_3844del	G1262RfsX3	2
CDK12	Pathogenic	3795_3873del	P1266CfsX23	1
CDK12	Pathogenic	4056_4134del	L1353VfsX23	1
CDK12	Pathogenic	4355dup	T1454NfsX50	1

CDK12	Pathogenic	446del	G149EfsX18	1
CDK12	Pathogenic	466G>T	E156X	1
CDK12	Pathogenic	522del	R175GfsX16	1
CDK12	Pathogenic	585del	D196TfsX142	1
CDK12	Pathogenic	604C>T	R202X	1
CDK12	Pathogenic	957C>G	Y319X	1
CDK12	Pathogenic	2947C>T	R983X	1
CDK12	VUS	2249_2251del	G750Del	1
CDK12	VUS	2261C>A	A754D	2
CDK12	VUS	2303C>T	P768L	1
CDK12	VUS	2635G>A	G879R	1
CDK12	VUS	2713G>C	E905Q	1
CDK12	VUS	2725G>C	G909R	1
CDK12	VUS	2932T>C	Y978H	1
CDK12	VUS	2962T>C	F988L	1
CDK12	VUS	3145C>A	R1049S	1
CDK12	VUS	3308-4A>G	?	1
CDK12	VUS	3310C>A	L1104I	1
CDK12	VUS	334C>A	R112S	1
CDK12	VUS	3584C>T	T1195M	1
CDK12	VUS	3797C>T	P1266L	1
CDK12	VUS	4313C>G	T1438S	1
CDK12	VUS	478G>A	D160N	1
CDK12	VUS	659C>G	S220C	1
CDK12	Likely pathogenic	141del	K48NfsX9	1
CDK12	Likely pathogenic	1886del	P629LfsX26	1
CDK12	Likely pathogenic	2576A>G	D859G	1
CDK12	Likely pathogenic	2609+2T>A	?	1
CDK12	Likely pathogenic	2635G>A	G879R	1
CDK12	Likely pathogenic	4028_4063del	G1343_D1354Del	1
CDK12	Likely pathogenic	585del	D196TfsX142	1
CDK12	Pathogenic	3518_130283519insTGCCCCAG AGGAGTCTTTGAAGGAACC AATCCATCAGTGCCCTGAC GGAAGCTACTTCCCAGCAG CAGGACTCAGAGACCAT	M1173IfsX53	1
CHEK2	Pathogenic	151del	Q51SfsX10	1

CHEK2	Pathogenic	1309G>A	E437K	1
CHEK2	Pathogenic	1338_1362del	Y447VfsX2	1
CHEK2	Pathogenic	1504G>C	A502P	1
CHEK2	Pathogenic	1556C>T	T519M	2
CHEK2	Pathogenic	1755_*85del	?	1
CHEK2	Pathogenic	188del	L63X	1
CHEK2	Pathogenic	248_319+8del	?	1
CHEK2	Pathogenic	596dup	Y199X	1
CHEK2	Pathogenic	722-1G>T	?	1
CHEK2	Pathogenic	813-1G>T	?	2
CHEK2	Pathogenic	copy number=0,99	LOH	1
CHEK2	VUS	1039A>G	M347V	1
CHEK2	VUS	1081C>T	R361C	1
CHEK2	VUS	1091T>C	I364T	1
CHEK2	VUS	1180G>A	E394K	1
CHEK2	VUS	1205A>G	E402G	1
CHEK2	VUS	1246A>G	I416V	1
CHEK2	VUS	1246A>G	K416E	4
CHEK2	VUS	1345C>T	R449C	1
CHEK2	VUS	1550G>A	R517H	1
CHEK2	VUS	1685G>T	R562L	5
CHEK2	VUS	1696C>T	R566C	1
CHEK2	VUS	1711G>A	E571K	1
CHEK2	VUS	196G>A	V66M	1
CHEK2	VUS	231_260del	D77_E86Del	1
CHEK2	VUS	246_260del	D82_E86Del	1
CHEK2	VUS	431G>A	R144Q	1
CHEK2	VUS	449-5T>A	?	8
CHEK2	VUS	542C>A	T181K	1
CHEK2	VUS	569C>T	A190V	1
CHEK2	VUS	580G>T	G194C	1
CHEK2	VUS	599T>C	I200T	1
CHEK2	VUS	671G>A	R224H	1
CHEK2	VUS	698C>T	A233V	1
CHEK2	VUS	844G>A	E282K	1
CHEK2	VUS	869C>T	A290V	1
CHEK2	Likely pathogenic	1180G>A	E394K	1
CHEK2	VUS	231_260del	D77_E86Del	1
